# A robust method for designing multistable systems by embedding bistable subsystems

**DOI:** 10.1038/s41540-022-00220-1

**Published:** 2022-03-25

**Authors:** Siyuan Wu, Tianshou Zhou, Tianhai Tian

**Affiliations:** 1 School of Mathematics, Monash University, Melbourne, VIC Australia; 2 School of Mathematics and Statistics, Sun Yet-Sen University, Guangzhou, China

**Keywords:** Multistability, Regulatory networks

## Abstract

Although multistability is an important dynamic property of a wide range of complex systems, it is still a challenge to develop mathematical models for realising high order multistability using realistic regulatory mechanisms. To address this issue, we propose a robust method to develop multistable mathematical models by embedding bistable models together. Using the *GATA1-GATA2-PU.1* module in hematopoiesis as the test system, we first develop a tristable model based on two bistable models without any high cooperative coefficients, and then modify the tristable model based on experimentally determined mechanisms. The modified model successfully realises four stable steady states and accurately reflects a recent experimental observation showing four transcriptional states. In addition, we develop a stochastic model, and stochastic simulations successfully realise the experimental observations in single cells. These results suggest that the proposed method is a general approach to develop mathematical models for realising multistability and heterogeneity in complex systems.

## Introduction

Multistability is the characteristic of a system that exhibits two or more mutually exclusive stable states. This phenomenon has been observed in many different disciplines of science, including genetic regulatory networks^1–4^, cell signalling pathways^5–8^, metabolic networks^9^, ecosystems^10,11^, neuroscience^12^, laser systems^13,14^, and quantum systems^15^. When external and/or internal conditions change, the system may switch from one steady state to another either randomly by perturbations or in a desired way according to the control strategies. In recent years mathematical models with multistability have been developed for theoretical analysis and computer simulations, which shed light on the mechanisms that generate multistability and control the transition between steady states^16–19^.

As one of the important molecular systems showing multistability, hematopoiesis is a highly integrated developmental process that controls the proliferation, differentiation and maturation of hematopoietic stem cells (HSCs)^20,21^. HSCs have the features of self-renewal and multipotency as well as the ability to differentiate into multipotent progenitors (MPPs). Each of these cell types is regarded as a stable state of the multistable system. In addition, the formation of white and red blood cells is a dynamical process that transits a cell from one stable cell type to another. This process begins with the differentiation of HSCs and enters the main stage at which cells reach either common myeloid progenitors (CMPs) or common lymphoid progenitors (CLPs)^22,23^.

Transcription factors play a key role in controlling the process of blood cell lineage specification. Experimental studies have demonstrated that the genetic module *GATA1-PU.1* is a vital component for the fate commitment of CMPs between erythropoiesis and granulopoiesis^24,25^. HSCs are more likely to choose megakaryocyte/erythroid progenitors (MEPs) with high expression levels of *GATA1*^26^, or conversely to choose granulocyte/macrophage progenitors (GMPs) with high expression levels of *PU.1*^27^. In addition, the regulation between genes *GATA1* and *GATA2* is an essential driver of hematopoiesis^28^. Experimental studies suggested that *GATA2* and *GATA1* sequentially bind the same *cis*-elements, which is referred to as the GATA-switching^29,30^.

Mathematical modelling is a powerful tool to accurately describe the dynamics of hematopoiesis and to explore the regulatory mechanisms for controlling the transitions between different cell types^31–37^. For the *GATA1-PU.1* module, Hill equations with high cooperativity were initially used to realise tristability^38^. In addition, mathematical models have been proposed to achieve bistability in gene regulatory networks without any high cooperativity coefficients^39,40^. Bifurcation theory is also an efficient method to explore the mechanisms of *GATA1-PU.1* module^41^. We have proposed a mathematical model to realise the mechanisms of GATA-switching and designed an effective algorithm to realise tristability of mathematical models^42^. Moreover, the underlying mechanisms of how the stem/progenitor cells leave the stable steady states and commit to a specific lineage were also revealed with the assistance of mathematical models^43^. At the single cell level, the differentiation processes of embryonic stem cells were simulated by Langevin equations, which helped to identify potential transcriptional regulators of lineage decision and commitment^44^. Mathematical models have also been used to study the dynamical properties of diseases such as periodic haematological disorders^45^.

Although these attempts have realised tristability by using different assumptions, it is still a challenge to develop mathematical models to realise tristability using both the realistic regulatory mechanisms and experimental data. On the other hand, substantial research studies have been conducted to develop mathematical models for realising bistability properties^3,46–51^. Thus, the question is whether we can develop mathematical models with tristability or higher order of multistability by using bistable models. To address this issue, we propose a robust method to develop multistable models by embedding bistable models together. Using the *GATA1-GATA2-PU.1* module as a testing model, we develop a tristable model based on two systems that have no high cooperativity coefficients.

## Results

### Embedding method for designing multistable models

**Fig. 1:**
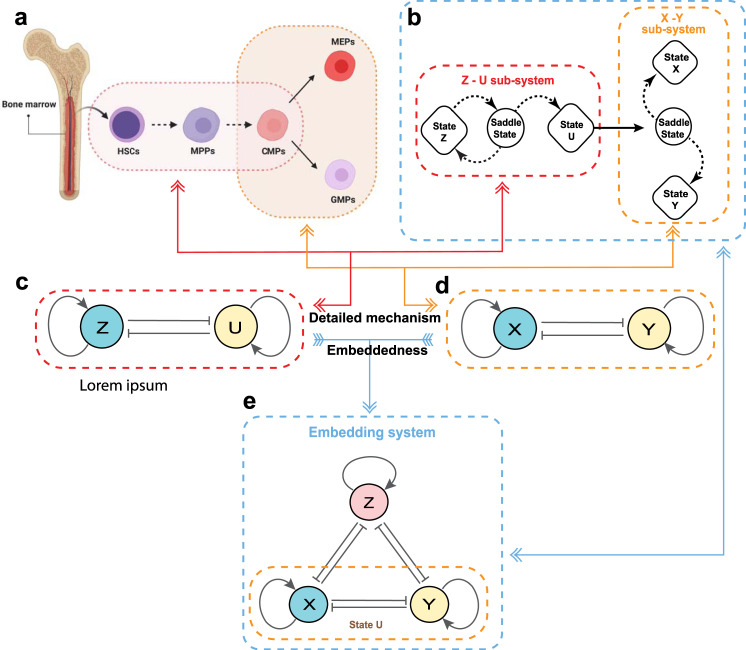
Methodology for developing multistable models by embedding two sub-systems with bistability together. **a** Brief flowchart of hematopoietic hierarchy that is created with BioRender.com. HSCs hematopoietic stem cells, MPPs multipotent progenitors, MEPs megakaryocyte-erythroid progenitors, GMPs granulocyte-macrophage progenitors. **b** The principle of embeddedness: *Z*-*U* module is the first bistable sub-system. Once this module crosses the saddle point from state *Z* to state *U*, it enters the *X*-*Y* sub-system that has two stable steady states *X* and *Y*, reaching either state *X* or state *Y* via the auxiliary state *U*. **c**, **d** The structure of two double-negative feedback loops with positive autoregulations, which is the mechanisms for bistable sub-systems in HSCs. **e** The structure and mathematical model of regulatory network after embeddedness. The *X*-*Y* sub-system is embedded into the state *U*.

The motivation of this work is to develop a mathematical model to realise the tristable property of the HSC genetic regulatory network in Fig. 1a based on experimental observations. Figure 1b, e illustrates the embedding method to couple two bistable modules in a network together, where ’ → ’ and ’⊣’ denote the activating and inhibiting regulations, respectively. Variable *U* in the first *Z*-*U* module is an auxiliary node, which is assumed to be *U* = *μX* + *δY*, where *μ* and *δ* are two positive parameters. When the system stays in the state with a high expression level of *Z* and a low level of *U*, the expression levels of *X* and *Y* are low. However, when the system has a low expression level of *Z* and a high level of *U*, the system triggers the second module *X*-*Y* to choose either a high level of *X* and a low level of *Y* or a low level of *X* and a high level of *Y*. In this way we realise the system with three stable states in which one of the three variables (namely *Z*, *X* or *Y*) is at the high expression state but the other two are at low expression states.

To demonstrate the effectiveness of the proposed embedding method, we use the toggle switch network as the test system^52^. This network consists of two genes that form a double negative feedback loop and is modelled by the following equations with parameter space Θ_1_ = {*a* = 0.2, *b* = 4, *c* = 3}, given by

1 $$\begin{array}{l}\frac{dz}{dt}={{{{\mathcal{F}}}}}_{1}(z,u,{{{{{\Theta }}}}}_{1},t)=0.2+\frac{4}{1+{u}^{3}}-z,\\ \frac{du}{dt}={{{{\mathcal{F}}}}}_{2}(z,u,{{{{{\Theta }}}}}_{1},t)=0.2+\frac{4}{1+{z}^{3}}-u.\end{array}$$

It is assumed that the first *Z*-*U* module follows model (1) and the second *X*-*Y* module satisfies the same model with same parameter space Θ_1_, but different variables *x* and *y*, given by

2 $$\begin{array}{l}\frac{dx}{dt}={{{{\mathcal{G}}}}}_{1}(x,y,{{{{{\Theta }}}}}_{1},t)=0.2+\frac{4}{1+{y}^{3}}-x,\\ \frac{dy}{dt}={{{{\mathcal{G}}}}}_{2}(x,y,{{{{{\Theta}}}}}_{1},t)=0.2+\frac{4}{1+{x}^{3}}-y.\end{array}$$

Now we embed these two sub-systems together using $u={{{\mathcal{H}}}}(x,y)=x+y$. Since gene *z* is negatively regulated by gene *u* in the sub-system (1), and *u* is a function of genes *x* and *y*, the expressions of genes *x* and *y* are also negatively regulated by gene *z* in the new embedding model. Then the non-linear vector fields ${{{{\mathcal{G}}}}}_{1,2}(x,y,{{{{{\Theta }}}}}_{1},t)$ are transformed into new non-linear vector fields ${{{{\mathcal{R}}}}}_{1,2}(x,y,z,{{{{{\Theta }}}}}_{1},t)$, respectively, which include genes *x*, *y* and *z* from two sub-systems with negative regulations from gene *z* to genes *x* and *y*. Therefore, the new model with three variables is given by

3 $$\begin{array}{l}\frac{dx}{dt}={{{{\mathcal{R}}}}}_{1}(x,y,z,{{{{{\Theta }}}}}_{1},t)=0.2+\frac{4}{(1+{y}^{3})(1+{z}^{3})}-x,\\ \frac{dy}{dt}={{{{\mathcal{R}}}}}_{w}(x,y,z,{{{{{\Theta }}}}}_{1},t)=0.2+\frac{4}{(1+{x}^{3})(1+{z}^{3})}-y,\\ \frac{dz}{dt}={{{{\mathcal{F}}}}}_{1}(z,u=x+y,{{{{{\Theta }}}}}_{1},t)=0.2+\frac{4}{1+{(x+y)}^{3}}-z.\end{array}$$

**Fig. 2:**
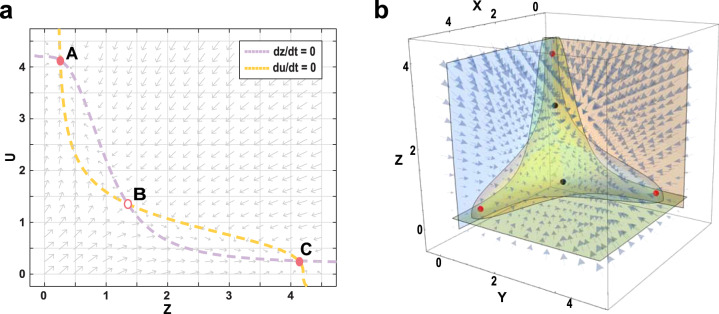
Realisation of tristability by embedding two bistable sub-systems. **a** The phase plane of the toggle switch sub-system (1) with bistability (A and B: stable steady states, C: saddle state). **b** The 3D phase portrait of the embedded system (3) with tristability (Three red points: stable steady states; two black points: saddle states).

Figure 2a shows the phase plane of the toggle switch sub-system (1) with bistability properties, and Fig. 2b provides the 3D phase portrait of the embedded model (3) with three stable steady states. The embedded model successfully realised the tristability, which validates our embedding method for developing mathematical models with multistability.

### Bistable models for ***G****ATA1-PU.1* and GATA-switching modules

For the two double-negative feedback loops with positive autoregulation in Fig. 1c, d, we next develop two mathematical models for the *Z*-*U* module (13) and *X*-*Y* module (14). These two models have the same structure but with different model parameters. Theorem 1 shows that there are five possible non-negative equilibria in these models. Theorem 2 indicates that two steady states located on the axis are stable under the given conditions. In addition, Theorem 3 gives the conditions under which two possible steady states located out of the axis are stable (see *Methods*).

We further search for stable steady states of the model with randomly sampled parameters. Supplementary Table 1 gives three types of bistable steady states. However, we have not found any parameter samples to realise tristability. To test robustness properties, we conduct perturbation tests by examining the bistable property of the model with slightly changed model parameters^53,54^. Our computational results demonstrate that a perturbed bistable model with one stable steady state located on the axis but another located off the axis can be found for a model with two stable steady states located on the axis (see Supplementary Table 2). These results suggest that the developed model has very good robustness properties in terms of parameter variations.

**Fig. 3:**
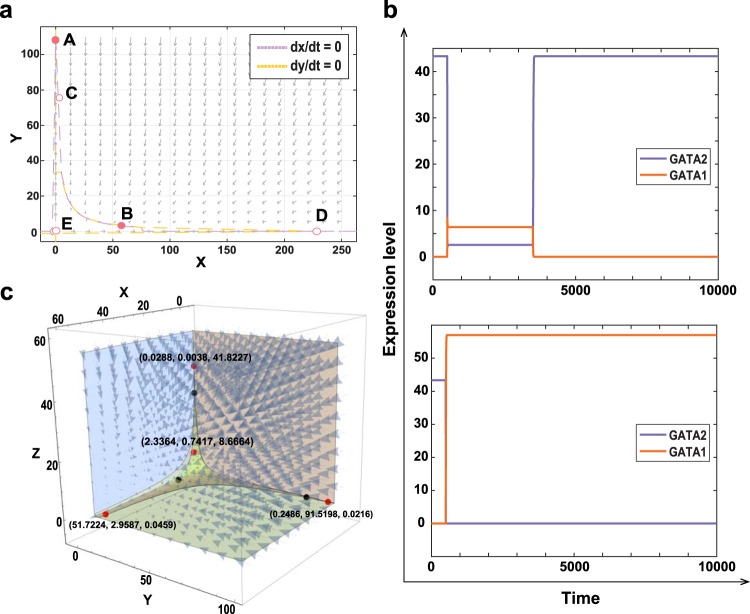
Realisation of tristability by embedding two bistable sub-systems in hematopoiesis. **a** Phase plane of the *GATA1-PU.1* module showing the bistable property of the proposed model, where A and B are stable steady states; C, D and E are saddle states. **b** Simulations of GATA-switching of model (5). Upper panel: An unsuccessful switching with a small value of ${k}_{0}^{* }$ due to the displacement of *GATA2* not being enough for cells to leave the HSCs state (*Z* state); Lower panel: A successful switching with sufficient displacement of *GATA2* by using a large value of ${k}_{0}^{* }$. Cells leave the HSCs state and enter the *U* state. **c** The 3D phase portrait of the modified embedding model (6) with k^*^ = 0. Four red points are stable steady states, while the three black points are saddle states.

We next use the approximate Bayesian computation (ABC) rejection algorithm^55,56^ to estimate model parameters based on the experimental data for erythroiesis and granulopoiesis^21^. We first estimate parameters in the *X*-*Y* module that describes regulations between genes *GATA1* and *PU.1*(14). It is assumed that the prior distribution of each parameter is a uniform distribution over the interval [0, 100]. The distance between experimental data and simulations is measured by

$$\rho ({{{\bf{X}}}},{{{\bf{{X}}}^{* }}})=\mathop{\sum }\limits_{i=1}^{m}[| {x}_{i}-{x}_{i}^{* }| +| {y}_{i}-{y}_{i}^{* }| ],$$

where (*x*_*i*_, *y*_*i*_) and (${x}_{i}^{* },{y}_{i}^{* }$) are the observed data and simulated data for genes (*X*, *Y*), respectively. Supplementary Table 3 gives the estimated parameters of this module. Figure 3a shows that the phase plane of the *GATA1-PU.1* sub-system based on estimated parameters, which shows that this system is bistable.

Regarding the *Z*-*U* module (13) that describes the regulation of GATA-switching, to be consistent with the module structure, we first assume that *GATA1* and *GATA2* form a double negative feedback module with autoregulations, and will modify this assumption later based on the experimentally observed mechanism. Here the data of the auxiliary variable *U* is the sum of *GATA1* and *PU.1*. Supplementary Table 4 gives the estimated parameters of the *Z*-*U* module.

An experimental study has identified *GATA2* at chromatin sites in early-stage erythroblasts^28^, when expression levels of *GATA1* increase as erythropoiesis progresses, *GATA1* displaces *GATA2* from chromatin sites. To describe the mechanism of GATA-switching, we introduce an additional rate constant k^*^ over a time interval [*t*_1_, *t*_2_] for the displacement rate of *GATA2* proteins during the process of GATA-switching, given by

4 $$\begin{array}{l}{k}^{* }=\left\{\begin{array}{ll}{k}_{0}^{* }\quad &t\in [{t}_{1},{t}_{2}],\\ 0\quad &\,{{\mbox{otherwise}}}\,.\end{array}\right.\end{array}$$

Since the displacement of GATA2 protein increasing, the concentration of GATA1 proteins around the binding site will increase proportionally to k^*^. Hence, we use rate ψk^*^z for the increase of *GATA1* during GATA-switching, where *ψ* is a control parameter to adjust the availability of *GATA1* proteins around chromatin sites. Then the GATA-switching module is modelled by

5 $$\begin{array}{l}\frac{dz}{dt}=\frac{{a}_{1}z}{1+{b}_{1}z}\frac{1}{1+{b}_{2}u}-{k}_{1}z-{k}^{* }z,\\ \frac{du}{dt}=\frac{{c}_{1}u}{1+{d}_{1}u}\frac{1}{1+{d}_{2}z}-{k}_{2}u+\psi {k}^{* }z,\end{array}$$

where *z* and *u* are expression levels of *GATA2* and *GATA1*, respectively. Note that the bistability property of this module is realised by model (5) using k^*^ = 0. Figure 3b gives two simulations for an unsuccessful switching and a successful switching. It is assumed that the GATA-switching occurs over the interval [*t*_1_, *t*_2_] = [500, 3500]. Simulations show that an adequate displacement of *GATA2* is the key to achieve GATA-switching using a relatively large value of ${k}_{0}^{* }\le 1$.

### Tristable model of the GATA1-GATA2-PU.1 network

After successfully realising the bistability in double-negative feedback loops with positive autoregulation, we next incorporate the *GATA1-PU.1* regulatory module into the GATA-switching module to realise the tristability of HSC differentiation. We use expression levels of *GATA1* in the GATA-switching module to represent total levels of *GATA1* plus *PU.1*, and embed these two modules together (18) (see Theorems 4–6 in *Methods* for more details). The model parameters have the same values as the corresponding parameters in the *Z*-*U* module or the *X*-*Y* module. Supplementary Fig. 1 gives the 3D phase portrait of the embedded system, which shows that the embedding model faithfully realises three stable steady states, which also suggests that the proposed embedding method is a robust approach to develop high order multistable models based on bistable models.

As mentioned in the previous subsection, the GATA-switching module is not a perfect double-negative feedback loop. In fact, experimental studies suggest that *GATA2* moderately simulates the expression of gene *GATA1*^57^. Thus we make a modification to model (18) by adding the term d^*^z in the first equation to represent a weak positive regulation from *GATA2* to *GATA1*. In addition, to avoid zero basal gene expression levels, we add a constant to each equation of the proposed model (18). The modified model is given by,

6 $$\begin{array}{l}\frac{dx}{dt}=\frac{{\alpha }_{0}\,+\,{\alpha }_{1}x}{1\,+\,{\beta }_{1}x}\frac{1}{1\,+\,{\beta }_{2}y}\frac{1\,+\,{d}^{* }z}{1\,+\,{d}_{2}z}-{k}_{3}x+\psi {k}^{* }z,\\ \frac{dy}{dt}=\frac{{\gamma }_{0}\,+\,{\gamma }_{1}y}{1\,+\,{\sigma }_{1}y}\frac{1}{1\,+\,{\sigma }_{2}x}\frac{1}{1\,+\,{d}_{2}z}-{k}_{4}y,\\ \frac{dz}{dt}=\frac{{a}_{0}\,+\,{a}_{1}z}{1\,+\,{b}_{1}z}\frac{1}{1\,+\,{b}_{2}(x\,+\,y)}-{k}_{1}z-{k}^{* }z,\end{array}$$

where *x*, *y*, *z* represent expression levels of genes *GATA1*, *GATA2* and *PU.1*, respectively. The values of *α*_0_, *γ*_0_, *a*_0_ and d^*^ are carefully selected so that the model simulation still matches experimental data and the model has at least three stable steady states (see Supplementary Table 5). Figure 3c gives the 3D phase portrait of system (6) with k^*^ = 0. Using estimated parameters (see Supplementary Tables 3–5), the modified system (6) actually achieves quad-stability. In three stable states, one of the three genes has high expression levels but the other two have low expression levels. The fourth stable state has low expression levels (2.3364, 0.7417, 8.6664) of the three genes. In fact, these are exact four transcriptional states that have been observed in experimental studies, namely a *PU*.1^*high*^*Gata*1/2^*low*^ state (P1H); a *Gata*1^*high*^*GATA*2/*PU*.1^*low*^ state (G1H); a *Gata*2^*high*^*GATA*1/*PU*.1^*low*^ state (G2H); and a state with low expression of all three genes (LES CMP)^21^. Compared with existing modelling studies, our embedding model (6) successfully realises the state with low expression levels of all three genes.

Note that the embedding model is based on the assumption of GATA-switching, namely the exchange of *GATA1* for *GATA2* at the chromatin site, which controls the expression of genes *GATA1* and *GATA2*. However, a low level of *GATA2* at the chromatin site does not mean the total level of *GATA2* in cells is also low. This may be the reason for the difference between the simulated state *Gata*1^*high*^*GATA*2/*PU*.1^*low*^ state (G1H) (namely only *GATA1* has high expression) and the experimentally observed state *Gata*1/2^*high*^*PU*.1^*low*^ state (G1/2H) (namely both *GATA1* and *GATA2* have high expression levels)^21^.

### Stochastic model for realising heterogeneity

**Fig. 4:**
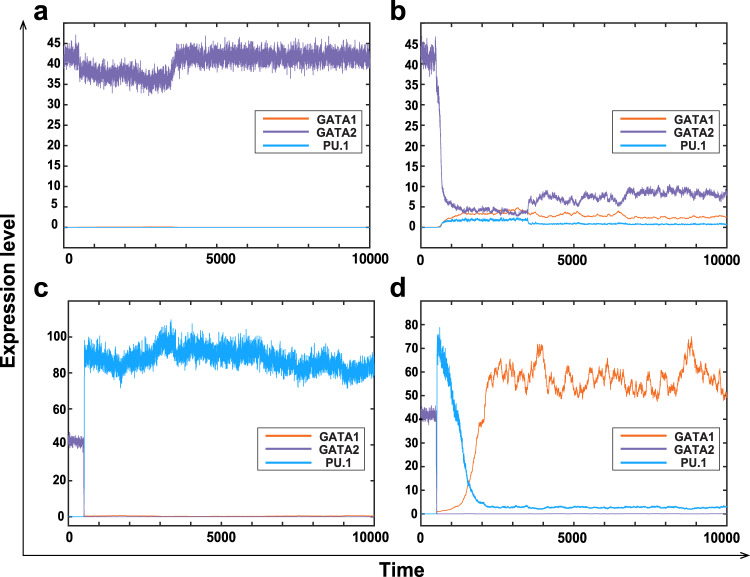
Stochastic simulations showing four stable states that correspond to the experimentally observed four different states. **a** Simulation of unsuccessful GATA switching that makes the cell stay at the HSC state, which is the G2H state. **b** Simulation of unsuccessful GATA switching but the cell enters the state with low expression of all three genes, which is the LES CMP state. **c** Simulation of successful switching that leads to the GMP state with high expression levels of *PU.1*, which is the P1H state. **d** Simulation of successful switching that leads to the MEP state with high expression levels of *GATA1*, which is the G1H state.

Although the modified embedding model has successfully realised the quad-stability properties, this deterministic model cannot describe the heterogeneity in the cell fate commitment. Thus, the next question is whether we can use a stochastic model to realise experimental data showing different gene expression levels in single cells^21^. To answer this question, we propose a stochastic differential equations model in Itô form to describe the functions of noise during the cell lineage specification, given by (7)

7 $$\begin{array}{l}dX(t)=\left[\frac{{\alpha }_{0}\,+\,{\alpha }_{1}X(t)}{1\,+\,{\beta }_{1}X(t)}\frac{1}{1\,+\,{\beta }_{2}Y(t)}\frac{1\,+\,{d}^{* }Z(t)}{1\,+\,{d}_{2}Z(t)}-{k}_{3}X(t)+\psi {k}^{* }Z(t)\right]dt+[{\omega }_{1}({k}_{3}X(t)+\psi {k}^{* }Z(t))]d{W}_{t}^{1},\\ dY(t)=\left[\frac{{\gamma }_{0}+{\gamma }_{1}Y(t)}{1\,+\,{\sigma }_{1}Y(t)}\frac{1}{1\,+\,{\sigma }_{2}X(t)}\frac{1}{1\,+\,{d}_{2}Z(t)}-{k}_{4}Y(t)\right]dt+[{\omega }_{2}{k}_{4}Y(t)]d{W}_{t}^{2},\\ dZ(t)=\left[\frac{{a}_{0}+{a}_{1}Z(t)}{1\,+\,{b}_{1}Z(t)}\frac{1}{\,1\,+\,{b}_{2}(X(t)\,+\,Y(t))}-{k}_{1}Z(t)-{k}^{* }Z(t)\right]dt+[{\omega }_{3}({k}_{1}+{k}^{* })Z(t)]d{W}_{t}^{3},\end{array}$$

where ${W}_{t}^{1}$, ${W}_{t}^{2}$ and ${W}_{t}^{3}$ are three independent Wiener processes whose increment is a Gaussian random variable Δ*W*_*t*_ = *W*(*t* + Δ*t*) − *W*(*t*) ~ *N*(0, Δ*t*), and *ω*_1_, *ω*_2_ and *ω*_3_ represent noise strengths. The reason for selecting Itô form is to maintain the mean of the stochastic system (7) as the corresponding deterministic system (6). To test the influence of GATA-switching on determining the transitions between different states, we introduce noise to coefficient k^*^ and consequently to the three degradation processes in the model. We use the semi-implicit Euler method to simulate the proposed model^58^. Figure 4 provides four stochastic simulations for four different types of cell fate commitments with model parameters ${k}_{0}^{* }=0.52$, *ψ* = 0.0005, *ω*_1_ = 0.04, and *ω*_2_ = *ω*_3_ = 0.08. Figure 4a, b shows two simulations of unsuccessful GATA switching when the displacement of *GATA2* is not sufficient. However, a sufficient displacement of *GATA2* can trigger successful GATA switching, which leads to either the GMP state with high expression levels of PU.1 in Fig. 4c or the MEP state with high expression levels of *GATA1* in Fig. 4d.

**Fig. 5:**
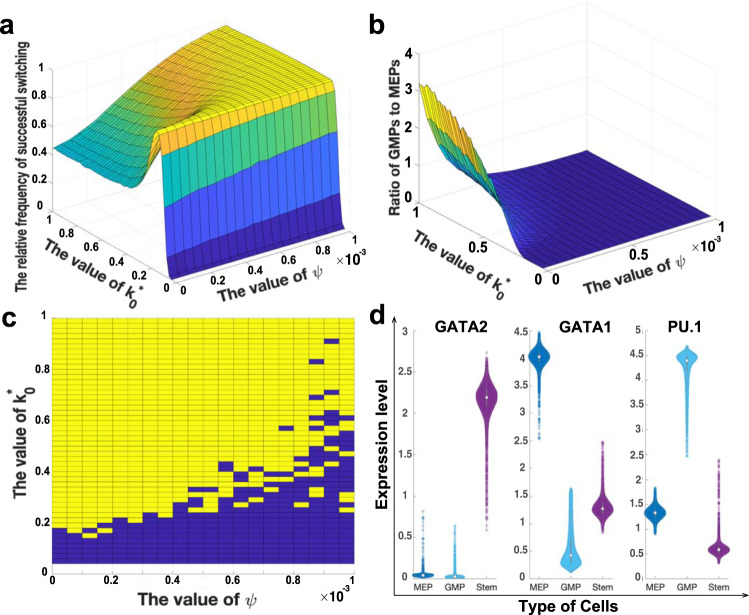
Distributions of different cell types derived from stochastic simulations. **a** Frequencies of cells having successful switching for each set of parameters $({k}_{0}^{* },\psi )$. **b** Ratios of GMP cells to MEP cells when cells have successfully switched in **a** for each set of parameters $({k}_{0}^{* },\psi )$. **c** Parameter sets of $({k}_{0}^{* },\psi )$ that generate stochastic simulations with four steady states as shown in Fig. 4 (yellow part) or with two or three states (blue part). **d** Violin plots of natural log normalised (expression level per cell +1) distributions for three genes in different cell states derived from stochastic simulations with parameters ${k}_{0}^{* }=0.52$ and *ψ* = 0.0005.

To examine the heterogeneity of hematopoiesis with different displacement rates ${k}_{0}^{* }$ and *ψ* together, we generate 20,000 stochastic simulations for each set of ${k}_{0}^{* }$ and *ψ* values over the range of [0.04, 1] and [0, 0.001], respectively. The ranges of ${k}_{0}^{* }$ and *ψ* are determined by numerical testing. If all stochastic simulations move to a single stable state for the given ${k}_{0}^{* }$ and *ψ* values, we change the lower bound and/or upper bound of the value range in order that simulations may move to different stable states for the given ${k}_{0}^{* }$ and *ψ* values. To show the boundary of parameter space, we also keep certain sets of parameter values with which simulations move to one specific stable state. Figure 5a gives proportions of simulations that have successful switching in 20,000 simulations. When the value of ${k}_{0}^{* }$ is between 0.1 and 0.2, the displacement speed of *GATA2* is low, which gives limited relief of negative regulation to *PU.1*, but *GATA1* increases gradually due to GATA-switching and weak positive regulation from *GATA2* to *GATA1*. Thus nearly all cells choose the MEP state with high expression levels of *GATA1*. However, if the value of ${k}_{0}^{* }$ is larger, the negative regulation from *GATA2* to *PU.1* is eliminated quickly, thus the competition between *GATA1* and *PU.1* will lead cells to different lineages. When the value of ${k}_{0}^{* }$ is relatively large but the value of *ψ* is relatively small, the increase of *GATA1* is slow due to the smaller value of *ψ* in GATA-switching. However, the negative regulation from GATA2 to PU.1 declines rapidly due to the larger value of ${k}_{0}^{* }$. Thus, Fig. 5b shows that the combination of larger ${k}_{0}^{* }$ and smaller *ψ* values allows more cells to move to the GMP lineage with high expression level of *PU.1*. If there is no winner in the competition between *GATA1* and *PU.1*, the cell then moves to the state with low expression levels of three genes (namely LE3G). Figure 5c shows that, when the value of ${k}_{0}^{* }$ is larger than 0.2, there are four types of simulations as shown in Fig. 5 for a set of ${k}_{0}^{* }$ and *ψ* values. We use a MATLAB package^59^ to give the violin plot for the expression distributions of three genes in three different cellular states. The violin plot is a combination of a box plot and a kernel density plot that illustrates data peaks. The violin plots in Fig. 5d match the experimental observations very well^21^.

Regarding the size of basins of attraction, we first calculate the distances between the stable states and saddle points in Fig. 3c, which are given in Supplementary Table 6. The minimal distance between the G1H state and three saddle points is much larger than the minimal distances of the other three stable states to the saddle points, which suggests that the size of basin of attraction for the G1H state is larger than those of the other three stable states. In addition, we observe the variability of stable states in 20,000 stochastic simulations. Supplementary Table 7 shows that the variations of GATA1 in the G1H state are much larger than those of the other two genes when having high expression levels.

We also study the relative frequency of LE3G state. Supplementary Fig. 2 shows that, for a fixed value of parameter *ψ*, the frequency increases as the value of ${k}_{0}^{* }$ increases. In addition, for a fixed value of ${k}_{0}^{* }$, the frequency decreases as the value of *ψ* increases. The variation of parameter *ψ* is much more important than that of parameter ${k}_{0}^{* }$. For the simulations showing in Fig. 5d, the frequency is 0.1080 with ${k}_{0}^{* }=0.52$ and *ψ* = 0.0005. Figure 5d and Supplementary Fig. 2 suggest that more cells remain in the LE3G or P1H (GMP) state if *GATA2* leaves the chromatin site fast (i.e. a large ${k}_{0}^{* }$ value) and the expression of *GATA1* is slow (i.e. a small *ψ* value). However, if the expression of *GATA1* is fast (i.e. a large *ψ* value), more cells will transit to G1H (MEP) state and the frequency of the LE3G state is low, which is consistent with the results in a recent study^60^.

## Discussions

Inspired by Waddington’s epigenetic landscape model, we assume that a multistable system makes a series of binary decisions for the selection of multiple evolutionary pathways. Compared with modelling studies for multistable networks, it is relatively easy to develop models with bistability and there is a rich literature for studying bistable networks. Thus, our proposed embedding method is an effective approach to develop multistable models based on well-studied models with bistable properties. In addition, using cell fate commitments in hematopoiesis as the test problem, we have successfully realised tristability in the *GATA-PU.1* module by embedding two bistable modules together. More importantly, by modifying the model using experimentally determined regulatory mechanisms, the developed model successfully realises four stable states that have been observed in a recent experimental study^21^.

In this study the stable states are achieved by a model without high cooperativity (i.e. Hill coefficient *n* = 1). Recently, the dynamics of toggle triad with self-activations have attracted much attention^60,61^. Mathematical models with high cooperativity have been developed to achieve pentastable, namely a hybrid *X*/*Y* state with high *X*, high *Y* and low *Z*. We tried to realise pentastability by using our proposed model with high cooperativity (*n* = 2 or 3), but numerical tests were not successful. Thus, high cooperativity in self-activation may be essential to realise pentastable. This is an interesting problem that will be the topic of further studies.

Despite the assumption of a binary choice in each sub-module, the developed model is able to realise a rich variety of dynamics. Our research suggests that, depending on the properties of bistable systems, the embedding model of two bistable modules may have more than three stable steady states. In addition, using the embedding method in Fig. 1, the state *U* is not a meta-stable state but actually disappears from the system. Simulations show that, when the system leaves the high *GATA2* expression state due to GATA-switching, genes *GATA1* and *PU.1* begin to increase their expression levels. Each stochastic simulation will reach one of the steady states with either high *GATA1* levels or high *PU.1* levels or return to the stem cell state. These simulations are consistent with the CLOUD-HSPC model in which differentiation is a process of uncommitted cells in transitory states that gradually acquire uni-lineage priming^62–64^. In addition, stochastic simulations demonstrate that noise plays a key role in determining different differentiation pathways.

This work uses differential equation models to determine stable steady states and then employs corresponding stochastic models to realise the functions of noise. However, experimental studies have shown that gene expression is a bursting process. The challenge is how to determine conditions for realising the multistable properties in stochastic models with bursting processes. In addition, hematopoiesis is a process to produce all mature blood cells. This is an ideal test system to develop mathematical models with multistable dynamics. An interesting question is how to embed more modules with more transcription factors to develop mathematical models with more stable steady states. All these issues will be interesting topics of further research.

## Methods

### Embedding method to couple models together

We propose a framework to model regulatory networks with multiple stable steady states based on the embedding of sub-systems with less stable steady states. It is assumed that we need to study a regulatory network that consists of two regulatory modules. The first module has genes *X*_*i*_, and it is modelled by the following equation

8 $$\frac{d{X}_{i}}{dt}={{{{\mathcal{F}}}}}_{i}({X}_{1},{X}_{2},\cdots \,,{X}_{n},{X}_{n+1},\cdots \,,{X}_{n+N},{{{{{\Theta }}}}}_{1},t)$$

for *i* = 1, 2, ⋯ , *n* + *N*, where Θ_1_ includes model parameters of ${{{{\mathcal{F}}}}}_{i}$. The second module has the following model

9 $$\frac{d{Y}_{j}}{dt}={{{{\mathcal{G}}}}}_{j}({Y}_{1},{Y}_{2},\cdots \,,{Y}_{m},{{{{{\Theta }}}}}_{2},t)$$

for *j* = 1, 2, ⋯ , *m*, where Θ_2_ includes model parameters of ${{{{\mathcal{G}}}}}_{j}$. In these two models, ${{{\mathcal{F}}}}({{{\bf{X}}}},{{{{{\Theta }}}}}_{1},t)$ and ${{{\mathcal{G}}}}({{{\bf{Y}}}},{{{{{\Theta }}}}}_{2},t)$ are non-linear vector fields. To develop mathematical models with more stable steady states, we propose an embedding method by assuming that *X*_*n*+*k*_ (*k* = 1, . . . , *N*) are functions of variables *Y*_1_, *Y*_2_, ⋯ , *Y*_*m*_, given by

10 $${X}_{n+k}={{{{\mathcal{H}}}}}_{k}({Y}_{1},{Y}_{2},\cdots \,,{Y}_{m}).$$

In this way, we obtain an embedding system

11 $$\frac{d{{{\bf{W}}}}}{dt}={{{\bf{F}}}}({{{\bf{W}}}},{{{{{\Theta }}}}}^{* },t),$$

where **W** = (*X*_1_, *X*_2_, ⋯ , *X*_*n*_, *Y*_1_, *Y*_2_, ⋯ , *Y*_*m*_) represents all genes in the system, **F** denotes the embedding system from two modules with gene *X*_*i*_ and *Y*_*i*_ with function ${{{{\mathcal{H}}}}}_{k}$. In addition, Θ^*^ = Θ_1_ ∪ Θ_2_ is the model parameters space. This embedding system (11) consists of two components:

12 $$\begin{array}{l}\frac{d{X}_{i}}{dt}={{{{\mathcal{F}}}}}_{i}({X}_{1},{X}_{2},\cdots \,,{X}_{n},{{{{\mathcal{H}}}}}_{k}({Y}_{1},{Y}_{2},\cdots \,,{Y}_{m}),{{{\Theta }}}^{* },t),\\ \frac{d{Y}_{j}}{dt}={{{{\mathcal{R}}}}}_{j}({X}_{1},{X}_{2},\cdots \,,{X}_{n},{Y}_{1},{Y}_{2},\cdots \,,{Y}_{m},{{{\Theta }}}^{* },t)\end{array}$$

for *i* = 1, 2, ⋯ , *n*, *k* = 1, . . . , *N* and *j* = 1, 2, ⋯ , *m*. Since each *X*_*i*_ is regulated by the *X*_*n*+*k*_ (*k* = 1, . . . , *N*), and *X*_*n*+*k*_ are functions of *Y*_1_, *Y*_2_, ⋯ , *Y*_*m*_, the expressions of each gene *Y*_*j*_ is also regulated by *X*_*i*_ (*i* = 1, . . . , *n*). The non-linear vector field ${{{\mathcal{G}}}}({{{\bf{Y}}}},{{{{{\Theta }}}}}_{2},t)$ in Eq. (9) will then be transformed into a new non-linear vector field ${{{\mathcal{R}}}}({{{\bf{W}}}},{{{{{\Theta }}}}}^{* },t)$, which includes both genes *X*_*i*_ and *Y*_*i*_ from two sub-systems with their corresponding regulations. Note that this is a general idea to develop mathematical models with more stable steady states. Depending on the specific formalism and properties of sub-systems, the embedding system may have different results regarding multiple stable steady states with different conditions. In this study, we only focus on the systems with Shea-Ackers formalism^65^.

### Model development for bistability properties

We first develop a model for the network in Fig. 1c with bistability properties. Suppose that two sub-systems, namely the *Z*-*U* system and *X*-*Y* sub-system, have the same structure of a double-negative feedback loop and positive autoregulations. For the *Z*-*U* system, based on the formalism (8) with **X** = {*z*, *u*} and Θ_1_ = {*a*_1_, *b*_1_, *b*_2_, *c*_1_, *d*_1_, *d*_2_, *k*_1_, *k*_2_}, we propose the following model to describe the dynamics, given by

13 $$\begin{array}{l}\frac{dz}{dt}={{{{\mathcal{F}}}}}_{1}(z,u,{{{{{\Theta }}}}}_{1},t)=\frac{{a}_{1}z}{1+{b}_{1}z}\frac{1}{1+{b}_{2}u}-{k}_{1}z,\\ \frac{du}{dt}={{{{\mathcal{F}}}}}_{2}(z,u,{{{{{\Theta }}}}}_{1},t)=\frac{{c}_{1}u}{1+{d}_{1}u}\frac{1}{1+{d}_{2}z}-{k}_{2}u.\end{array}$$

Similarly, based on the formalism (9) with **Y** = {*x*, *y*} and Θ_2_ = {*α*_1_, *β*_1_, *β*_2_, *γ*_1_, *σ*_1_, *σ*_2_, *k*_3_, *k*_4_}, the dynamics of the *X* − *Y* subsystem is modelled by

14 $$\begin{array}{l}\frac{dx}{dt}={{{{\mathcal{G}}}}}_{1}(x,y,{{{{{\Theta }}}}}_{2},t)=\frac{{\alpha }_{1}x}{1+{\beta }_{1}x}\frac{1}{1+{\beta }_{2}y}-{k}_{3}x,\\ \frac{dy}{dt}={{{{\mathcal{G}}}}}_{2}(x,y,{{{{{\Theta }}}}}_{2},t)=\frac{{\gamma }_{1}y}{1+{\sigma }_{1}y}\frac{1}{1+{\sigma }_{2}x}-{k}_{4}y,\end{array}$$

where *x* and *y* are expression levels of genes *X* and *Y*, respectively; *α*_1_ and *γ*_1_ represent expression rates; *β*_1_, *β*_2_, *σ*_1_ and *σ*_2_ represent association rates of corresponding proteins to binding-sites; and *k*_3_ and *k*_4_ are self-degradation rates. The model of the *Z*-*U* subsystem has the same structure but may have different values of model parameters. To obtain the bistability, we establish the following theorems for our proposed models for these two sub-systems. Since they have the same structure, we only give the theorems for the *X*-*Y* sub-system.

#### Theorem 1

There are at most five sets of non-negative equilibria for model (14).

1. There are three equilibria: (0, 0), (*x*_*e*_, 0) and (0, *y*_*e*_), where ${x}_{e}=\frac{{\alpha }_{1}-{k}_{3}}{{k}_{3}{\beta }_{1}}$ and ${y}_{e}=\frac{{\gamma }_{1}-{k}_{4}}{{k}_{4}{\sigma }_{1}}$, if *α*_1_ > *k*_3_ and *γ*_1_ > *k*_4_.
2. There are two other equilibria: $({x}_{1}^{* },{y}_{1}^{* })$ and $({x}_{2}^{* },{y}_{2}^{* })$. If $-\frac{{{{\mathcal{B}}}}}{{{{\mathcal{A}}}}}\,>\, 0$, $\frac{{{{\mathcal{C}}}}}{{{{\mathcal{A}}}}} \,>\, 0$ and ${{{{\mathcal{B}}}}}^{2}-4{{{\mathcal{A}}}}{{{\mathcal{C}}}}\ge 0$, then ${x}_{1}^{* }$ and ${x}_{2}^{* }$ are positive real solutions of the following equation,
  15 $${{{\mathcal{A}}}}{m}^{2}+{{{\mathcal{B}}}}m+{{{\mathcal{C}}}}=0,$$
  where $$m={\beta }_{1}x,{{{\mathcal{A}}}}={A}_{1}{B}_{1}-{B}_{1},{{{\mathcal{B}}}}={A}_{1}-{B}_{1}-1+{A}_{1}{B}_{1}-{A}_{1}{B}_{2}+{A}_{2}{B}_{1}$$, $${{{\mathcal{C}}}}={A}_{1}+{A}_{2}-1-{A}_{1}{B}_{2}$$, $${A}_{1}=\frac{{\beta }_{2}}{{\sigma }_{1}},{A}_{2}=\frac{{\alpha }_{1}}{{k}_{3}},{B}_{1}=\frac{{\sigma }_{2}}{{\beta }_{1}}$$ and $${B}_{2}=\frac{{\gamma }_{1}}{{k}_{4}}$$.
3. To have positive values of ${y}_{1}^{* }$ and ${y}_{2}^{* }$, the following conditions should be satisfied,
  16 $${x}_{1,2}^{* }\, <\, \frac{{A}_{2}-1}{{\beta }_{1}}\,{{\mbox{or}}}\,{x}_{1,2}^{* } \,<\, \frac{{B}_{2}-1}{{\sigma }_{2}}.$$

Moreover, to study the bistability, it is necessary to establish conditions of stability/instability for each equilibrium state. We first give the following conditions for each equilibrium state that locates on an axis.

#### Theorem 2

The *X*-*Y* system has three equilibria: (0, 0), (*x*_*e*_, 0) and (0, *y*_*e*_).

1. The equilibrium state (0, 0) is unstable if *α*_1_ > *k*_3_ and *γ*_1_ > *k*_4_.
2. The equilibrium state (*x*_*e*_, 0) is stable if $\frac{{\gamma }_{1}}{1+{\sigma }_{2}{x}_{e}}\, < \,{k}_{4}$.
3. The equilibrium state (0, *y*_*e*_) is stable if $\frac{{\alpha }_{1}}{1+{\beta }_{2}{y}_{e}}\, < \,{k}_{3}$.

In addition, we give the following stable conditions for each equilibrium state that locates within the 2-dimensional positive real space.

#### Theorem 3

The positive equilibria $({x}_{1}^{* },{y}_{1}^{* })$ and $({x}_{2}^{* },{y}_{2}^{* })$ are stable if the following condition is satisfied.

17 $${\beta }_{1}{\sigma }_{1}{\eta }_{y}{\xi }_{x}-{\beta }_{2}{\sigma }_{2}{\theta }_{x}{\rho }_{y} \,>\, 0.$$

where *θ*_*x*_ = 1 + *β*_1_*x*, *η*_*y*_ = 1 + *β*_2_*y*, *ρ*_*y*_ = 1 + *σ*_1_*y* and *ξ*_*x*_ = 1 + *σ*_2_*x*.

In summary, Theorem 1 gives the existence conditions of the equilibria for our proposed two-node systems. Theorems 2 and 3 provide the necessary conditions for stability properties of these equilibria. According to these theorems, we can easily check whether two-node systems have bistability based on generated samples of model parameters. The proofs of these theorems are given in Supplementary Notes.

### Perturbation analysis of bistable models

We have proved that systems (13) and (14) have bistable steady states under the conditions in Theorems 2 or 3. Next we use the random search method to find the model parameters with which the system has bistable steady states. We first generate a sample for each model parameter from the uniform distribution over the interval [0, *A*] and then test whether the system with the sampled parameters satisfies the conditions in Theorems 2 or 3. If the conditions are satisfied, we solve nonlinear equations of the system to find the steady states. We test different values of *A* and find that the system has bistable steady states when *A* = 10. To find more types of bistable states, we test 10000 sets of parameters from the uniform distribution over the interval [0, 10]. Supplementary Table 1 gives three types of bistable steady states, namely Case 1: (*x*_*e*_, 0) and (0, *y*_*e*_); Case 2: (*x*_*e*_, 0) and $({x}_{1}^{* },{y}_{1}^{* })$; and Case 3: (0, *y*_*e*_) and $({x}_{2}^{* },{y}_{2}^{* }).$ All stable states in case 1 are located on the coordinate axis. We add a perturbation to each estimated coefficient *c* as c^*^ = [*ε* × (*P* − 0.5) + 1] × *c*, where *P* is a uniformly distributed random variable over the interval [0, 1], and *ε* is the strength of perturbation. Supplementary Table 2 shows that the two other cases of bistability can be obtained by the perturbed coefficients from Case 1.

### Model development for tristability properties

The mathematical model for the network of three genes is formed by embedding the *X*-*Y* system into the *Z*-*U* system as shown in Fig. 1d. For simplicity, let $u={{{\mathcal{H}}}}(x,y)\,$ = *x* + *y*. Since gene *z* is negatively regulated by gene *u* in sub-system (13), and *u* is a function of genes *x* and *y*, the expressions of genes *x* and *y* are also negatively regulated by gene *z* in the new embedding model. The non-linear vector fields ${{{{\mathcal{G}}}}}_{1,2}(x,y,{{{{{\Theta }}}}}_{1},t)$ are then transformed into new non-linear vector fields ${{{{\mathcal{R}}}}}_{1,2}(x,y,z,{{{{{\Theta }}}}}^{* },t)$, respectively, which include genes *x*, *y* and *z* from two sub-systems with negative regulations from gene *z* to genes *x* and *y*. Using the embedding method (12) and sub-system models ((13), (14)), we obtain the following model to describe the embedded *X*-*Y*-*Z* system,

18 $$\begin{array}{l}\frac{dx}{dt}={{{{\mathcal{R}}}}}_{1}(x,y,z,{{{{{\Theta }}}}}^{* },t)=\frac{{\alpha }_{1}x}{1+{\beta }_{1}x}\frac{1}{1+{\beta }_{2}y}\frac{1}{1+{d}_{2}z}-{k}_{3}x,\\ \frac{dy}{dt}={{{{\mathcal{R}}}}}_{2}(x,y,z,{{{{{\Theta }}}}}^{* },t)=\frac{{\gamma }_{1}y}{1+{\sigma }_{1}y}\frac{1}{1+{\sigma }_{2}x}\frac{1}{1+{d}_{2}z}-{k}_{4}y,\\ \frac{dz}{dt}={{{{\mathcal{F}}}}}_{1}(z,u=x+y,{{{{{\Theta }}}}}^{* },t)=\frac{{a}_{1}z}{1+{b}_{1}z}\frac{1}{1+{b}_{2}(x+y)}-{k}_{1}z.\end{array}$$

To verify the tristability of model (18), we give the following conditions for existence of the equilibria and necessary conditions for stability properties of these equilibria.

#### Theorem 4

1. If (*x*_*e*_, 0) and (0, *y*_*e*_) are equilibria of *X*-*Y* sub-system and (*z*_*e*_, 0) is a equilibrium state of *Z*-*U* sub-system, where ${x}_{e}=\frac{{\alpha }_{1}-{k}_{3}}{{k}_{3}{\beta }_{1}}$, ${y}_{e}=\frac{{\gamma }_{1}-{k}_{4}}{{k}_{4}{\sigma }_{1}}$ and ${z}_{e}=\frac{{a}_{1}-{k}_{1}}{{k}_{1}{b}_{1}}$, then (*x*_*e*_, 0, 0), (0, *y*_*e*_, 0) and (0, 0, *z*_*e*_) are three equilibria of the embedding system (18).
2. If $({x}_{1}^{* },{y}_{1}^{* })$ and $({x}_{2}^{* },{y}_{2}^{* })$ are two positive equilibria of *X*-*Y* system as stated in Theorem 1, then $({x}_{1}^{* },{y}_{1}^{* },0)$ and $({x}_{2}^{* },{y}_{2}^{* },0)$ are still two equilibria of the embedding system (18).

This theorem shows that existence conditions of equilibria in the embedded system are the same as those of two-node sub-systems. Thus, the information of two-node sub-systems can be directly applied to the embedded system. For each equilibrium state located on the axis, we give the following conditions of stability.

#### Theorem 5

If (*x*_*e*_, 0) and (0, *y*_*e*_) are both stable states of *X*-*Y* system and (*z*_*e*_, 0) is a stable state of *Z*-*U* system.

1. The equilibrium state (*x*_*e*_, 0, 0) is stable if $\frac{{a}_{1}}{1+{b}_{2}{x}_{e}} \,<\, {k}_{1}$.
2. The equilibrium state (0, *y*_*e*_, 0) is stable if $\frac{{a}_{1}}{1+{b}_{2}{y}_{e}} \,<\, {k}_{1}$.
3. The equilibrium state (0, 0, *z*_*e*_) is stable if $\frac{{\alpha }_{1}}{1+{d}_{2}{z}_{e}} \,<\, {k}_{3}$ and $\frac{{\gamma }_{1}}{1+{d}_{2}{z}_{e}} \,<\, {k}_{4}$.

In addition, we give the following stable conditions for each equilibrium state that locates within the 3-dimensional positive real space.

#### Theorem 6

Suppose (x^*^, y^*^) is a stable state of *X*-*Y* system, then the equilibrium state (x^*^, y^*^, 0) is also a stable state of the *X*-*Y*-*Z* system if

19 $$\frac{{a}_{1}}{1+{b}_{2}({x}^{* }+{y}^{* })} < {k}_{1}.$$

Theorems 5 and 6 describe the necessary conditions for stability properties of the equilibria in the embedding *X*-*Y*-*Z* system. By applying these theorems, we can further constrain the estimated parameters obtained from two-node systems so that the embedding system can achieve tristability. The proofs of Theorems 4–6 are given in Supplementary Notes.

## Supplementary information

Supplementary Information

## Data Availability

No datasets were generated during the current study. The experimental data for erythroiesis and granulopoiesis that support the parameter estimation of this study are available in the published paper at https://www.nature.com/articles/s41586-020-2432-4^21^.

## Code availability

The code used to perform the analyses presented in the current study is available from the corresponding author on reasonable request.
