# Supplementary material for: A robust method for designing multistable systems by embedding bistable subsystems
Source: NPJ Syst Biol Appl. 2022 Mar 25;8:10. doi: 10.1038/s41540-022-00220-1 (PMC8956579; doi:10.1038/s41540-022-00220-1)
Supplement: Supplementary file 1 — Supplementary Information [file 41540_2022_220_MOESM1_ESM.pdf]

Supplementary Information

A robust method for designing multistable systems by embedding bistable subsystems

Siyuan Wu, Tianshou Zhou, and Tianhai Tian

January 19, 2022

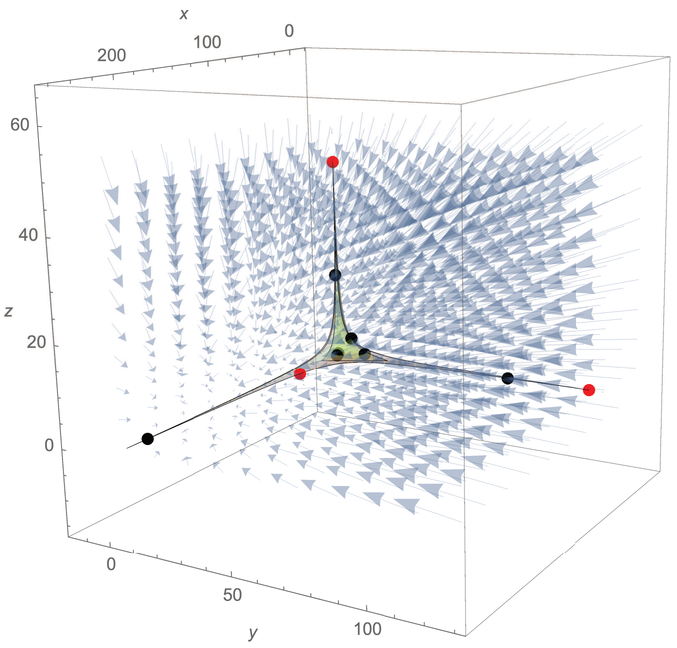

Supplementary Figure 1: **The 3D phase portrait of the embedded system (18).** Based on the experimental data, the proposed model successfully realise the tristability properties, with the same parameter values presented in the **Supplementary Table 3** and **Supplementary Table 4**. Red points: stable steady states; Black points: saddle states.

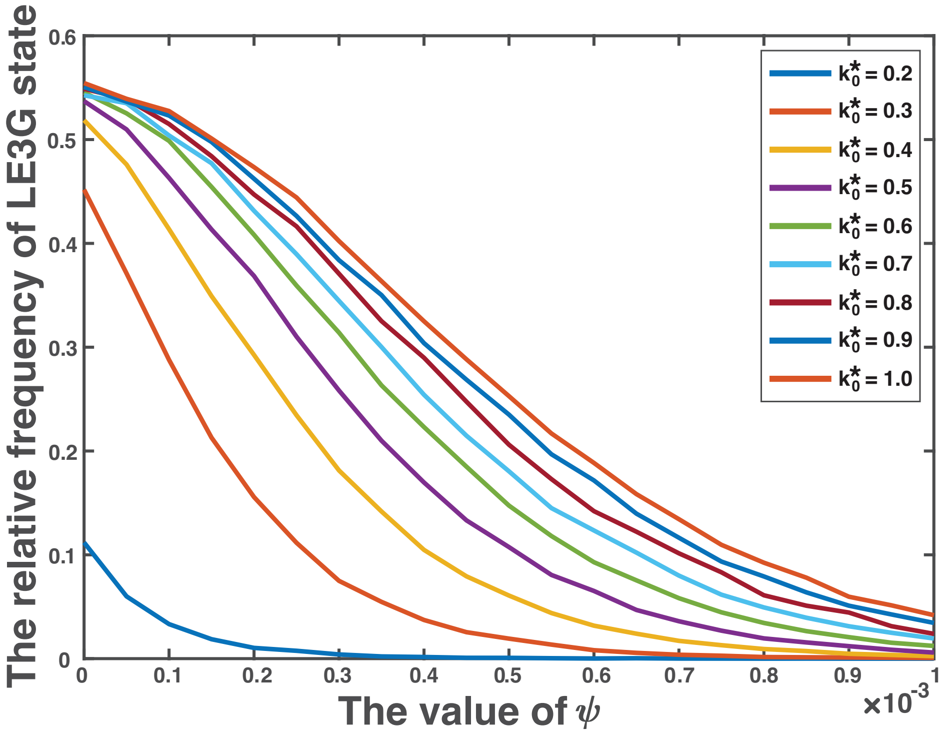

Supplementary Figure 2: **The relative frequency of LE3G state with different values of k_0^*.**

	α_1	β_1	β_2	γ_1	σ_1	σ_2	k_3	k_4	Equilibrium State	Characteristic	Stability
**Type 1**	4.0252	1.1393	8.5862	7.5202	2.1524	4.6084	0.5924	0.6515	(0.0000, 4.8982)	Nodal Sink	**Stable**
									(1.6517, 0.1581)	Saddle Point	**Unstable**
									(5.0862, 0.0000)	Nodal Sink	**Stable**
**Type 2**	8.0486	2.0932	0.3926	8.4293	1.1974	4.5864	1.2308	0.3546	(0.0000, 19.0173)	Nodal Sink	**Stable**
									(0.2556, 8.3039)	Saddle Point	**Unstable**
									(1.1683, 2.2871)	Nodal Sink	**Stable**
									(2.6463, 0.0000)	Saddle Point	**Unstable**
**Type 3**	8.6817	3.6357	5.7823	2.7531	1.3007	0.5855	1.1452	1.4741	(0.0000, 0.6671)	Saddle Poing	**Unstable**
									(0.3034, 0.4505)	Nodal Sink	**Stable**
									(1.2241, 0.0676)	Saddle Point	**Unstable**
									(1.8101, 0.0000)	Nodal Sink	**Stable**

Supplementary Table 1: **Three types of the bistable states that locate at different positions.** Type 1: two stable states are located on the axis; Type 2 and Type 3, one of the stable states is located on an axis but the other is are located out of the axis.

	α_1	β_1	β_2	γ_1	σ_1	σ_2	k_3	k_4	Equilibrium State	Characteristic	Stability
**Type 1**	4.0252	1.1393	8.5862	7.5202	2.1524	4.6084	0.5924	0.6515	(0.0000, 4.8982)	Nodal Sink	**Stable**
									(1.6517, 0.1581)	Saddle Point	**Unstable**
									(5.0862, 0.0000)	Nodal Sink	**Stable**
**Perturbed case 1**	4.2582	0.3682	1.7541	11.8512	3.8062	1.4729	0.6912	0.4352	(0.0000, 6.8918)	Nodal Sink	**Stable**
									(2.5527, 1.2403)	Saddle Point	**Unstable**
									(9.2824, 0.2249)	Nodal Sink	**Stable**
									(14.0157, 0.0000)	Saddle Point	**Unstable**
**Perturbed case 2**	4.9263	1.6689	9.6312	0.8750	3.1029	0.8549	0.5510	0.2678	(0.0000, 0.7307)	Saddle Point	**Unstable**
									(0.2915, 0.5206)	Nodal Sink	**Stable**
									(1.0317, 0.2372)	Saddle Point	**Unstable**
									(4.7580, 0.0000)	Nodal Sink	**Stable**

Supplementary Table 2: **Perturbation analysis with strength** $\varepsilon = 1.8$**.** Type 1 is the Type 1 case in **Supplementary Table** 1. Perturbed cases 1 and 2 are obtained from Type 1 by perturbing the model parameters. In these two cases, one the stable state is located on an axis but the other is located out of the axis.

$\alpha_1 = 15.665$	$\beta_1 = 0.4263$	$\beta_2 = 0.9047$	$k_3 = 0.1587$
$\gamma_1 = 89.4$	$\sigma_1 = 1.0724$	$\sigma_2 = 0.4535$	$k_4 = 0.752$

Supplementary Table 3: **Estimated model parameter values for module X-Y.**

$a_1 = 16.5$	$b_1 = 0.6024$	$b_2 = 1.1$	$k_1 = 0.6090$
$c_1 = 4.2934$	$d_1 = 0.3340$	$d_2 = 3.6$	$k_2 = 0.2143$

Supplementary Table 4: **Estimated model parameter values for module Z-U.**

$\alpha_0 = 0.045$	$\gamma_0 = 0.1$	$a_0 = 1$	$d^* = 0.01$

Supplementary Table 5: **Estimated additional model parameter values for modified model.**

		Unstable States		
Stable States		**(0.3170, 0.0100, 31.4818)**	**(0.6179, 78.1130, 0.0265)**	**(13.3231, 2.7597, 0.9070)**
**G1H**	**(51.7224, 2.9587, 0.0459)**	60.3277	90.8837	38.4095
**P1H**	**(0.2486, 91.5198, 0.0216)**	96.7667	13.4119	89.7222
**G2H**	**(0.0288, 0.0038, 41.8227)**	10.3449	88.5907	43.1095
**LE3G**	**(2.3364, 0.7417, 8.6664)**	22.9163	77.8712	13.6010

Supplementary Table 6: **Distances between four stable states and three saddle points shown in the phase portrait of Figure 3c.**

		G1H (MEP) State	P1H (GMP) State	G2H (HSC) State	LE3G State
**GATA1**	**Deterministic Solution**	**51.7224**	**0.2486**	**0.0288**	**2.3364**
	**Min**	34.8137	0.1344	0.0243	1.5030
	**Max**	77.0405	1.0789	0.0374	4.2895
**PU.1**	**Deterministic Solution**	**2.9587**	**91.5298**	**0.0038**	**0.7414**
	**Min**	1.6972	70.6865	0.0026	0.4675
	**Max**	4.3167	105.4327	0.0057	1.2441
**GATA2**	**Deterministic Solution**	**0.0459**	**0.0216**	**41.8227**	**8.6664**
	**Min**	0.0265	0.0180	36.5418	4.8823
	**Max**	0.0868	0.0351	47.7615	12.7441

Supplementary Table 7: **The expression variations in stochastic simulations around the four stable states of the corresponding deterministic model.** The deterministic solutions (*GATA1, PU.1, GATA2*) for G1H, P1H, G2H and LE3G states are (51.7224, 2.9587, 0.0459), (0.2486, 91.5298, 0.0216), (0.0288, 0.0038, 41.8227) and (2.3364, 0.7414, 8.6664), respectively (also shown in **Figure 3c**). The minimal/maximal expression levels of each gene are obtained from 20000 stochastic simulations for each state.

Supplementary Notes: Proofs of theorems 1 to 6

Theorem 1. *There are at most five sets of non-negative equilibria for the model of the X-Y system.*

1. *There are three equilibria:* $(0,0)$, $(x_e,0)$ *and* $(0,y_e)$, *where* $x_e = \frac{\alpha_1-k_3}{k_3\beta_1}$ *and* $y_e = \frac{\gamma_1-k_4}{k_4\sigma_1}$, *if* $\alpha_1 > k_3$ *and* $\gamma_1 > k_4$.
2. *There are two other equilibria:* (x_1^*, y_1^*) *and* (x_2^*, y_2^*). *If* $-\frac{\mathcal{B}}{\mathcal{A}} > 0$, $\frac{\mathcal{C}}{\mathcal{A}} > 0$ *and* $\mathcal{B}^2 - 4\mathcal{A}\mathcal{C} \geq 0$, *then* x_1^* *and* x_2^* *are positive real solutions of the following equation,*
$$\mathcal{A}m^2 + \mathcal{B}m + \mathcal{C} = 0, \tag{1}$$
where $m = \beta_1 x, \mathcal{A} = A_1B_1 - B_1, \mathcal{B} = A_1 - B_1 - 1 + A_1B_1 - A_1B_2 + A_2B_1$, $\mathcal{C} = A_1 + A_2 - 1 - A_1B_2$, $A_1 = \frac{\beta_2}{\sigma_1}, A_2 = \frac{\alpha_1}{k_3}, B_1 = \frac{\sigma_2}{\beta_1}$ *and* $B_2 = \frac{\gamma_1}{k_4}$.
3. *To have positive values of* y_1^* *and* y_2^*, *the following conditions should be satisfied,*
$$x_{1,2}^* < \frac{A_2 - 1}{\beta_1} \text{ or } x_{1,2}^* < \frac{B_2 - 1}{\sigma_2}. \tag{2}$$

Proof. Suppose the the equilibrium state exists, then we have

$$\frac{\alpha_1 x}{1+\beta_1 x}\frac{1}{1+\beta_2 y} - k_3 x = 0, \tag{3}$$
$$\frac{\gamma_1 y}{1+\sigma_1 y}\frac{1}{1+\sigma_2 x} - k_4 y = 0. \tag{4}$$

Then, we consider the following three cases.

1.
   - The trivial solution: $(0,0)$.
   - When the equilibrium state is $(x_e, 0)$, where $x_e \neq 0$. According to (3), we have
   $$x_e = \frac{\alpha_1 - k_3}{k_3\beta_1}. \tag{5}$$
   Since α_1, k_3 and β_1 are positive, we have the positive equilibrium solution if $\alpha_1 > k_3$.
   - When the equilibrium state is $(0, y_e)$, where $y_e \neq 0$. According to (4), we have
   $$y_e = \frac{\gamma_1 - k_4}{k_4\sigma_1}. \tag{6}$$
   Since γ_1, k_4 and σ_1 are positive, we have the positive equilibrium solution if $\gamma_1 > k_4$.

2. When the equilibria are (x_1^*, y_1^*) and (x_2^*, y_2^*), where all values here are not zero, according to (3) and (4), we have

$$\frac{\alpha_1}{(1+\beta_1 x)(1+\beta_2 y)} = k_3, \tag{7}$$

$$\frac{\gamma_1}{(1+\sigma_1 y)(1+\sigma_2 x)} = k_4. \tag{8}$$

Let $m = \beta_1 x$ and $n = \sigma_1 y$, we have

$$(1+m)(1+\frac{\beta_2}{\sigma_1}n) = \frac{\alpha_1}{k_3}, \tag{9}$$

$$(1+n)(1+\frac{\sigma_2}{\beta_1}m) = \frac{\gamma_1}{k_4}. \tag{10}$$

Let $A_1 = \frac{\beta_2}{\sigma_1}, A_2 = \frac{\alpha_1}{k_3}, B_1 = \frac{\sigma_2}{\beta_1}$ and $B_2 = \frac{\gamma_1}{k_4}$. Finally, we can get

$$(1+m)(1+A_1 n) = A_2, \tag{11}$$

$$(1+n)(1+B_1 m) = B_2. \tag{12}$$

From (11) and (12), we have

$$n = \frac{A_2 - 1 - m}{A_1 + A_1 m} = \frac{B_2 - 1 - B_1 m}{1 + B_1 m}. \tag{13}$$

That is,

$$(A_1B_1 - B_1)m^2 + (A_1 - B_1 - 1 + A_1B_1 - A_1B_2 + A_2B_1)m + (A_1 + A_2 - 1 - A_1B_2) = 0.$$

Let $\mathcal{A} = A_1B_1 - B_1$, $\mathcal{B} = A_1 - B_1 - 1 + A_1B_1 - A_1B_2 + A_2B_1$ and $\mathcal{C} = A_1 + A_2 - 1 - A_1B_2$. Then, we have the following quadratic function

$$\mathcal{A}m^2 + \mathcal{B}m + \mathcal{C} = 0. \tag{14}$$

(a) If $\Delta = \mathcal{B}^2 - 4\mathcal{A}\mathcal{C} = 0$, there is only one solution, namely $m = \frac{-\mathcal{B}}{2\mathcal{A}}$. Thus, the solution of $m = \frac{-\mathcal{B}}{2\mathcal{A}}$ is positive if $-\frac{\mathcal{B}}{\mathcal{A}} > 0$. Then we have

$$x_1^* = x_2^* = \frac{-\mathcal{B}}{2\beta_1\mathcal{A}} \text{ and } y_1^* = y_2^* = \frac{A_2 - 1 - \beta_1 x_{1,2}^*}{\beta_2(1+\beta_1 x_{1,2}^*)} = \frac{B_2 - 1 - \sigma_2 x_{1,2}^*}{\sigma_1(1+\sigma_2 x_{1,2}^*)}. \tag{15}$$

(b) If $\Delta > 0$, there are two distinct real solutions. If the following conditions are satisfied, we will have two distinct positive real solutions of m:

i. $m_1 + m_2 = -\frac{\mathcal{B}}{\mathcal{A}} > 0$,

ii. $m_1 m_2 = \frac{\mathcal{C}}{\mathcal{A}} > 0$,

iii. $\mathcal{B}^2 - 4\mathcal{A}\mathcal{C} > 0$

In this case, the solution of (14) is

$$m = \frac{-\mathcal{B} \pm \sqrt{\mathcal{B}^2 - 4\mathcal{A}\mathcal{C}}}{2\mathcal{A}} \tag{16}$$

the solution of n satisfies (13). Substitute $x^* = \frac{m}{\beta_1}$ and $y^* = \frac{n}{\sigma_1}$ into the solution, we have the solution of (x_1^*, y_1^*) and (x_2^*, y_2^*)

$$x_{1,2}^* = \frac{-\mathcal{B} \pm \sqrt{\mathcal{B}^2 - 4\mathcal{A}\mathcal{C}}}{2\beta_1\mathcal{A}} \tag{17}$$

$$y_{1,2}^* = \frac{A_2 - 1 - \beta_1 x_{1,2}^*}{\beta_2(1 + \beta_1 x_{1,2}^*)} = \frac{B_2 - 1 - \sigma_2 x_{1,2}^*}{\sigma_1(1 + \sigma_2 x_{1,2}^*)}. \tag{18}$$

3. From the proof of both part 2(a) and 2(b), if $\Delta \geq 0$, we have the expression of $y_{1,2}^*$, as follows

$$y_{1,2}^* = \frac{A_2 - 1 - \beta_1 x_{1,2}^*}{\beta_2(1 + \beta_1 x_{1,2}^*)} = \frac{B_2 - 1 - \sigma_2 x_{1,2}^*}{\sigma_1(1 + \sigma_2 x_{1,2}^*)}. \tag{19}$$

It is clear that, if $x_{1,2}^* < \frac{A_2-1}{\beta_1}$ or $x_{1,2}^* < \frac{B_2-1}{\sigma_2}$, the corresponding value of $y_{1,2}^*$ is positive as well.

□

Theorem 2. *The model of the X-Y system has three equilibria:* $(0,0)$, $(x_e, 0)$ *and* $(0, y_e)$.

1. *The equilibrium state* $(0,0)$ *is unstable if* $\alpha_1 > k_3$ *and* $\gamma_1 > k_4$.

2. *The equilibrium state* $(x_e, 0)$ *is stable if* $\frac{\gamma_1}{1+\sigma_2 x_e} < k_4$.

3. *The equilibrium state* $(0, y_e)$ *is stable if* $\frac{\alpha_1}{1+\beta_2 y_e} < k_3$.

Proof. The Jacobian matrix $\mathbf{J}_{(x,y)} = [\mathbf{J}_{ij}]_{2\times 2}$ of the X-Y system is defined by

$$\mathbf{J}_{11} = \frac{\partial \dot{x}}{\partial x}|_{(x,y)=(x_0,y_0)} = \frac{\alpha_1}{(1+\beta_1 x_0)^2}\frac{1}{1+\beta_2 y_0} - k_3, \tag{20}$$

$$\mathbf{J}_{12} = \frac{\partial \dot{x}}{\partial y}|_{(x,y)=(x_0,y_0)} = \frac{\alpha_1 x_0}{1+\beta_1 x_0}\frac{-\beta_2}{(1+\beta_2 y_0)^2}, \tag{21}$$

$$\mathbf{J}_{21} = \frac{\partial \dot{y}}{\partial x}|_{(x,y)=(x_0,y_0)} = \frac{\gamma_1 y_0}{1+\sigma_1 y_0}\frac{-\sigma_2}{(1+\sigma_2 x_0)^2}, \tag{22}$$

$$\mathbf{J}_{22} = \frac{\partial \dot{y}}{\partial y}|_{(x,y)=(x_0,y_0)} = \frac{\gamma_1}{(1+\sigma_1 y_0)^2}\frac{1}{1+\sigma_2 x_0} - k_4. \tag{23}$$

(1). The Jacobian matrix at the equilibrium state $(0,0)$ is

$$\mathbf{J}_{(0,0)} = \begin{bmatrix} \alpha_1 - k_3 & 0 \\ 0 & \gamma_1 - k_4 \end{bmatrix}. \tag{24}$$

The eigenvalues of the Jacobian matrix are $\lambda_1 = \alpha_1 - k_3$ and $\lambda_2 = \gamma_1 - k_4$. Obviously, the equilibrium state $(0,0)$ is unstable if any one of the following conditions are satisfied

$$\alpha_1 > k_3, \quad \gamma_1 > k_4. \tag{25}$$

Notice that the above conditions are also the existence conditions for equilibria $(x_e, 0)$ and $(0, y_e)$ which has been proved in **Theorem 1**. In this case, we prove that when $(0,0)$ is an unstable state, there exist two positive equilibria $(x_e, 0)$ and $(0, y_e)$.

(2). The Jacobian matrix at the equilibrium state $(x_e, 0) = (\frac{\alpha_1 - k_3}{k_3\beta_1}, 0)$ is

$$\mathbf{J}_{(x_e,0)} = \begin{bmatrix} \frac{\alpha_1}{(1+\beta_1 x_e)^2} - k_3 & \frac{-\alpha_1\beta_2 x_e}{1+\beta_1 x_e} \\ 0 & \frac{\gamma_1}{1+\sigma_2 x_e} - k_4 \end{bmatrix}. \tag{26}$$

When $x_e \neq 0$, the eigenvalues of the Jacobian matrix are

$$\lambda_1 = \frac{\alpha_1}{(1+\beta_1 x_e)^2} - k_3 = \frac{\alpha_1}{(1+\beta_1 x_e)^2} - \frac{\alpha_1}{1+\beta_1 x_e} = \frac{-\alpha_1\beta_1 x_e}{(1+\beta_1 x_e)^2}, \tag{27}$$

$$\lambda_2 = \frac{\gamma_1}{1+\sigma_2 x_e} - k_4 \tag{28}$$

It is clear that $\lambda_1 < 0$. Thus, this equilibrium state is stable if

$$\frac{\gamma_1}{1+\sigma_2 x_e} < k_4. \tag{29}$$

(3). The Jacobian matrix at the equilibrium state $(0, y_e) = (0, \frac{\gamma_1 - k_4}{k_4\sigma_1})$ is

$$\mathbf{J}_{(0,y_e)} = \begin{bmatrix} \frac{\alpha_1}{1+\beta_2 y_e} - k_3 & 0 \\ \frac{-\gamma_1\sigma_2 y_e}{1+\sigma_1 y_e} & \frac{\gamma_1}{(1+\sigma_1 y_e)^2} - k_4 \end{bmatrix}. \tag{30}$$

When $y_e \neq 0$, the eigenvalues of the Jacobian matrix are

$$\lambda_1 = \frac{\alpha_1}{1+\beta_2 y_e} - k_3, \tag{31}$$

$$\lambda_2 = \frac{\gamma_1}{(1+\sigma_1 y_e)^2} - k_4 = \frac{\gamma_1}{(1+\sigma_1 y_e)^2} - \frac{\gamma_1}{1+\sigma_1 y_e} = \frac{-\gamma_1\sigma_1 y_e}{(1+\sigma_1 y_e)^2} \tag{32}$$

It is clear that $\lambda_2 < 0$. Thus, this equilibrium state is stable if

$$\frac{\alpha_1}{1+\beta_2 y_e} < k_3. \tag{33}$$

□

Theorem 3. *The positive equilibria (x_1^*, y_1^*) and (x_2^*, y_2^*) are stable if the following condition is satisfied.*

$$\beta_1\sigma_1\eta_y\xi_x - \beta_2\sigma_2\theta_x\rho_y > 0, \tag{34}$$

where $\theta_x = 1+\beta_1 x$, $\eta_y = 1+\beta_2 y$, $\rho_y = 1+\sigma_1 y$ and $\xi_x = 1+\sigma_2 x$.

Proof. The Jacobian matrix at the equilibrium state (x, y) is

$$\mathbf{J}_{(x,y)} = \begin{bmatrix} \frac{\alpha_1}{(1+\beta_1 x)^2(1+\beta_2 y)} - k_3 & \frac{-\alpha_1\beta_2 x}{(1+\beta_1 x)(1+\beta_2 y)^2} \\ \frac{-\gamma_1\sigma_2 y}{(1+\sigma_1 y)(1+\sigma_2 x)^2} & \frac{\gamma_1}{(1+\sigma_1 y)^2(1+\sigma_2 x)} - k_4 \end{bmatrix} \tag{35}$$

When x and y are not zero, we substitute (7) and (8) into the Jacobian matrix (35). Then, we have

$$\mathbf{J}_{(x,y)} = \begin{bmatrix} \frac{\alpha_1(1-\theta_x)}{\theta_x^2\eta_y} & \frac{-\alpha_1\beta_2 x}{\theta_x\eta_y^2} \\ \frac{-\gamma_1\sigma_2 y}{\rho_y\xi_x^2} & \frac{\gamma_1(1-\rho_y)}{\rho_y^2\xi_x} \end{bmatrix}. \tag{36}$$

The eigenvalues of the Jacobian matrix (36) are

$$\lambda = \frac{-\Phi \pm \sqrt{\Phi^2 - \tau}}{2\theta_x^2\eta_y^2\rho_y^2\xi_x^2}, \tag{37}$$

where

$$\Phi = \eta_y\xi_x(\rho_y^2\xi_x\alpha_1\beta_1 x + \theta_x^2\eta_y\gamma_1\sigma_1 y), \tag{38}$$

$$\tau = 4\theta_x^2\eta_y^2\rho_y^2\xi_x^2\alpha_1\gamma_1 xy(\beta_1\sigma_1\eta_y\xi_x - \beta_2\sigma_2\theta_x\rho_y). \tag{39}$$

For both (x_1^*, y_1^*) and (x_2^*, y_2^*) to be stable, it requires that both eigenvalues are negative or have negative real parts. Thus, the stability conditions of (x_1^*, y_1^*) and (x_2^*, y_2^*) are $\Phi > 0$ and $\tau > 0$. Note that, $\Phi > 0$ is always true, since all values in condition (38) are positive. However, $\tau > 0$ if and only if $\beta_1\sigma_1\eta_{y^*}\xi_{x^*} - \beta_2\sigma_2\theta_{x^*}\rho_{y^*} > 0$. Thus, we have proved that if $\beta_1\sigma_1\eta_{y^*}\xi_{x^*} - \beta_2\sigma_2\theta_{x^*}\rho_{y^*} > 0$, the positive equilibria (x_1^*, y_1^*) and (x_2^*, y_2^*) are stable.
□

Theorem 4. *1. If $(x_e, 0)$ and $(0, y_e)$ are the equilibria of X-Y sub-system and $(z_e, 0)$ is a equilibrium state of Z-U sub-system, where $x_e = \frac{\alpha_1 - k_3}{k_3\beta_1}$, $y_e = \frac{\gamma_1 - k_4}{k_4\sigma_1}$ and $z_e = \frac{a_1 - k_1}{k_1 b_1}$, then $(x_e, 0, 0)$, $(0, y_e, 0)$ and $(0, 0, z_e)$ are three equilibria of the embedding X-Y-Z system.*

2. If (x_1^*, y_1^*) and (x_2^*, y_2^*) are two positive equilibria of X-Y system as stated in **Theorem 1**, *then $(x_1^*, y_1^*, 0)$ and $(x_2^*, y_2^*, 0)$ are still two equilibria of the embedding X-Y-Z system.*

Proof. From **Theorem 1**, we have proved that the Z-U system has the equilibrium state $(z_e, 0)$, where $z_e = \frac{a_1 - k_1}{k_1 b_1}$ if $a_1 > k_1$. Moreover, the X-Y system has the equilibria $(x_e, 0)$ and $(0, y_e)$, where $x_e = \frac{\alpha_1 - k_3}{k_3\beta_1}$ and $y_e = \frac{\gamma_1 - k_4}{k_4\sigma_1}$, if $\alpha_1 > k_3$ and $\gamma_1 > k_4$. Let us consider the X-Y-Z system. Suppose the equilibrium state exists. Then we have

$$\frac{\alpha_1 x}{1 + \beta_1 x}\frac{1}{1 + \beta_2 y}\frac{1}{1 + d_2 z} - k_3 x = 0, \tag{40}$$

$$\frac{\gamma_1 y}{1 + \sigma_1 y}\frac{1}{1 + \sigma_2 x}\frac{1}{1 + d_2 z} - k_4 y = 0, \tag{41}$$

$$\frac{a_1 z}{1 + b_1 z}\frac{1}{1 + b_2(x + y)} - k_1 z = 0. \tag{42}$$

1. (a) When $y = z = 0$, according to (40), we have

$$x_e = \frac{\alpha_1 - k_3}{k_3\beta_1}. \tag{43}$$

Since α_1, k_3 and β_1 are positive, we have the positive equilibrium solution if $\alpha_1 > k_3$.

(b) When $x = z = 0$, according to (41), we have

$$y_e = \frac{\gamma_1 - k_4}{k_4\sigma_1}. \tag{44}$$

Since γ_1, k_4 and σ_1 are positive, we have the positive equilibrium solution if $\gamma_1 > k_4$.

(c) When $x = y = 0$, according to (42), we have

$$z_e = \frac{a_1 - k_1}{k_1 b_1}. \tag{45}$$

Since a_1, k_1 and b_1 are positive, we have the positive equilibrium solution if $a_1 > k_1$.

2. When $z = 0$, the system will reduced to

$$\frac{\alpha_1 x}{1+\beta_1 x}\frac{1}{1+\beta_2 y} - k_3 x = 0, \tag{46}$$

$$\frac{\gamma_1 y}{1+\sigma_1 y}\frac{1}{1+\sigma_2 x} - k_4 y = 0, \tag{47}$$

where (46) and (47) are the same as equations (7) and (8) in the proof of **Theorem 1**.

It is clear to see that, for all cases, the conditions for the existence of these equilibria in the X-Y-Z system are the same as those in the two bistable sub-systems Z-U and X-Y. □

Theorem 5. *If $(x_e, 0)$ and $(0, y_e)$ are both stable states of X-Y system and $(z_e, 0)$ is a stable state of Z-U system.*

1. *The equilibrium state $(x_e, 0, 0)$ is stable if $\frac{a_1}{1+b_2 x_e} < k_1$.*
2. *The equilibrium state $(0, y_e, 0)$ is stable if $\frac{a_1}{1+b_2 y_e} < k_1$.*
3. *The equilibrium state $(0, 0, z_e)$ is stable if $\frac{\alpha_1}{1+d_2 z_e} < k_3$ and $\frac{\gamma_1}{1+d_2 z_e} < k_4$.*

Proof. The Jacobian matrix $\mathbf{J}_{(x,y,z)} = [\mathbf{J}_{ij}]_{3\times 3}$ of the X-Y-Z system is defined by

$$\mathbf{J}_{11} = \frac{\partial \dot{x}}{\partial x}\Big|_{(x,y,z)=(x_0,y_0,z_0)} = \frac{\alpha_1}{(1+\beta_1 x_0)^2}\frac{1}{1+\beta_2 y_0}\frac{1}{1+d_2 z_0} - k_3, \tag{48}$$

$$\mathbf{J}_{12} = \frac{\partial \dot{x}}{\partial y}\Big|_{(x,y,z)=(x_0,y_0,z_0)} = \frac{\alpha_1 x_0}{1+\beta_1 x_0}\frac{-\beta_2}{(1+\beta_2 y_0)^2}\frac{1}{1+d_2 z_0}, \tag{49}$$

$$\mathbf{J}_{13} = \frac{\partial \dot{x}}{\partial z}\Big|_{(x,y,z)=(x_0,y_0,z_0)} = \frac{\alpha_1 x_0}{1+\beta_1 x_0}\frac{1}{1+\beta_2 y_0}\frac{-d_2}{(1+d_2 z_0)^2}, \tag{50}$$

$$\mathbf{J}_{21} = \frac{\partial \dot{y}}{\partial x}\Big|_{(x,y,z)=(x_0,y_0,z_0)} = \frac{\gamma_1 y_0}{1+\sigma_1 y_0}\frac{-\sigma_2}{(1+\sigma_2 x_0)^2}\frac{1}{1+d_2 z_0}, \tag{51}$$

$$\mathbf{J}_{22} = \frac{\partial \dot{y}}{\partial y}\Big|_{(x,y,z)=(x_0,y_0,z_0)} = \frac{\gamma_1}{(1+\sigma_1 y_0)^2}\frac{1}{1+\sigma_2 x_0}\frac{1}{1+d_2 z_0} - k_4, \tag{52}$$

$$\mathbf{J}_{23} = \frac{\partial \dot{y}}{\partial z}\Big|_{(x,y,z)=(x_0,y_0,z_0)} = \frac{\gamma_1 y_0}{1+\sigma_1 y_0}\frac{1}{1+\sigma_2 x_0}\frac{-d_2}{(1+d_2 z_0)^2}, \tag{53}$$

$$\mathbf{J}_{31} = \frac{\partial \dot{z}}{\partial x}\Big|_{(x,y,z)=(x_0,y_0,z_0)} = \frac{a_1 z_0}{1+b_1 z_0}\frac{-b_2}{(1+b_2(x_0+y_0))^2}, \tag{54}$$

$$\mathbf{J}_{32} = \frac{\partial \dot{z}}{\partial y}\Big|_{(x,y,z)=(x_0,y_0,z_0)} = \frac{a_1 z_0}{1+b_1 z_0}\frac{-b_2}{(1+b_2(x_0+y_0))^2}, \tag{55}$$

$$\mathbf{J}_{33} = \frac{\partial \dot{z}}{\partial z}\Big|_{(x,y,z)=(x_0,y_0,z_0)} = \frac{a_1}{(1+b_1 z_0)^2}\frac{1}{1+b_2(x_0+y_0)} - k_1. \tag{56}$$

1. The Jacobian matrix at the equilibrium state $(x_e, 0, 0) = (\frac{\alpha_1 - k_3}{k_3\beta_1}, 0, 0)$ is

$$\mathbf{J}_{(x_e,0,0)} = \begin{bmatrix} \frac{\alpha_1}{(1+\beta_1 x_e)^2} - k_3 & \frac{-\alpha_1\beta_2 x_e}{1+\beta_1 x_e} & \frac{-\alpha_1 d_2 x_e}{1+\beta_1 x_e} \\ 0 & \frac{\gamma_1}{1+\sigma_2 x_e} - k_4 & 0 \\ 0 & 0 & \frac{a_1}{1+b_2 x_e} - k_1 \end{bmatrix} \tag{57}$$

When $x_e \neq 0$, the eigenvalues of the Jacobian matrix are

$$\lambda_1 = \frac{\alpha_1}{(1+\beta_1 x_e)^2} - k_3 = \frac{\alpha_1}{(1+\beta_1 x_e)^2} - \frac{\alpha_1}{1+\beta_1 x_e} = \frac{-\alpha_1\beta_1 x_e}{(1+\beta_1 x_e)^2}, \tag{58}$$

$$\lambda_2 = \frac{\gamma_1}{1+\sigma_2 x_e} - k_4, \tag{59}$$

$$\lambda_3 = \frac{a_1}{1+b_2 x_e} - k_1. \tag{60}$$

It is clear that $\lambda_1 < 0$ and $\lambda_2 < 0$ since $(x_e, 0) = (\frac{\alpha_1 - k_3}{k_3\beta_1}, 0)$ is a stable state of the X-Y system. Thus, this equilibrium state is stable if

$$\frac{a_1}{1+b_2 x_e} < k_1. \tag{61}$$

2. The Jacobian matrix at the equilibrium state $(0, y_e, 0) = (0, \frac{\gamma_1 - k_4}{k_4\sigma_1}, 0)$ is

$$\mathbf{J}_{(0,y_e,0)} = \begin{bmatrix} \frac{\alpha_1}{1+\beta_2 y_e} - k_3 & 0 & 0 \\ \frac{-\sigma_2\gamma_1 y_e}{1+\sigma_1 y_e} & \frac{\gamma_1}{(1+\sigma_1 y_e)^2} - k_4 & \frac{-d_2\gamma_1 y_e}{1+\sigma_1 y_e} \\ 0 & 0 & \frac{a_1}{1+b_2 y_e} - k_1 \end{bmatrix} \tag{62}$$

When $y_e \neq 0$, the eigenvalues of the Jacobian matrix are

$$\lambda_1 = \frac{\alpha_1}{1+\beta_2 y_e} - k_3, \tag{63}$$

$$\lambda_2 = \frac{\gamma_1}{(1+\sigma_1 y_e)^2} - k_4 = \frac{\gamma_1}{(1+\sigma_1 y_e)^2} - \frac{\gamma_1}{1+\sigma_1 y_e} = \frac{-\gamma_1\sigma_1 y_e}{(1+\sigma_1 y_e)^2}, \tag{64}$$

$$\lambda_3 = \frac{a_1}{1+b_2 y_e} - k_1. \tag{65}$$

It is clear that $\lambda_2 < 0$ and $\lambda_1 < 0$ since $(0, y_e) = (0, \frac{\gamma_1 - k_4}{k_4\sigma_1})$ is a stable state of the X-Y system. Thus, this equilibrium state is stable if

$$\frac{a_1}{1+b_2 y_e} < k_1. \tag{66}$$

3. The Jacobian matrix at the equilibrium state $(0, 0, z_e) = (0, 0, \frac{a_1 - k_1}{k_1 b_1})$ is

$$\mathbf{J}_{(0,0,z_e)} = \begin{bmatrix} \frac{\alpha_1}{1+d_2 z_e} - k_3 & 0 & 0 \\ 0 & \frac{\gamma_1}{1+d_2 z_e} - k_4 & 0 \\ \frac{-a_1 b_2 z_e}{1+b_1 z_e} & \frac{-a_1 b_2 z_e}{1+b_1 z_e} & \frac{a_1}{(1+b_1 z_e)^2} - k_1 \end{bmatrix} \tag{67}$$

When $z_e \neq 0$, the eigenvalues of the Jacobian matrix are

$$\lambda_1 = \frac{\alpha_1}{1 + d_2 z_e} - k_3, \tag{68}$$

$$\lambda_2 = \frac{\gamma_1}{1 + d_2 z_e} - k_4, \tag{69}$$

$$\lambda_3 = \frac{a_1}{(1 + b_1 z_e)^2} - k_1 = \frac{a_1}{(1 + b_1 z_e)^2} - \frac{a_1}{1 + b_1 z_e} = \frac{-a_1 b_1 z_e}{(1 + b_1 z_e)^2}. \tag{70}$$

(71)

It is clear that $\lambda_3 < 0$. Thus, this equilibrium state is stable if

$$\frac{\alpha_1}{1 + d_2 z_e} < k_3 \text{ and } \frac{\gamma_1}{1 + d_2 z_e} < k_4. \tag{72}$$

□

Theorem 6. *Suppose (x^*, y^*) is a stable state of X-Y system, then the equilibrium state $(x^*, y^*, 0)$ is also a stable state of the X-Y-Z system if*

$$\frac{a_1}{1 + b_2(x^* + y^*)} < k_1. \tag{73}$$

Proof. The Jacobian matrix of X-Y-Z system $\mathbf{J}_{(x,y,z)} = [\mathbf{J}_{ij}]_{3\times3}$ is defined in the proof of **Theorem 5**. Assume that x^* and y^* are both non-zero, then the Jacobian matrix at $(x^*, y^*, 0)$ is

$$\mathbf{J}_{(x^*,y^*,0)} = \begin{bmatrix} \frac{\alpha_1}{(1+\beta_1 x^*)^2(1+\beta_2 y^*)} - k_3 & -\frac{\alpha_1 \beta_2 x^*}{(1+\beta_1 x^*)(1+\beta_2 y^*)^2} & -\frac{\alpha_1 d_2 x^*}{(1+\beta_1 x^*)(1+\beta_2 y^*)} \\ -\frac{\gamma_1 \sigma_2 y^*}{(1+\sigma_1 y^*)(1+\sigma_2 x^*)^2} & \frac{\gamma_1}{(1+\sigma_1 y^*)^2(1+\sigma_2 x^*)} - k_4 & -\frac{\gamma_1 d_2 y^*}{(1+\sigma_1 y^*)(1+\sigma_2 x^*)} \\ 0 & 0 & \frac{a_1}{1+b_2(x^*+y^*)} - k_1 \end{bmatrix} \tag{74}$$

Substitute (7) and (8) into the Jacobian matrix (74). Since $\theta_x = 1 + \beta_1 x$, $\eta_y = 1 + \beta_2 y$, $\rho_y = 1 + \sigma_1 y$ and $\xi_x = 1 + \sigma_2 x$. Then, we have

$$\mathbf{J}_{(x^*,y^*,0)} = \begin{bmatrix} \frac{\alpha_1(1-\theta_x)}{\theta_x^2\eta_y} & -\frac{\alpha_1\beta_2 x^*}{\theta_x\eta_y^2} & -\frac{\alpha_1 d_2 x^*}{\theta_x\eta_y} \\ -\frac{\gamma_1\sigma_2 y^*}{\rho_y\xi_x^2} & \frac{\gamma_1(1-\rho_y)}{\rho_y^2\xi_x} & -\frac{\gamma_1 d_2 y^*}{\rho_y\xi_x} \\ 0 & 0 & \frac{a_1}{1+b_2(x^*+y^*)} - k_1 \end{bmatrix}. \tag{75}$$

The eigenvalues of the Jacobian matrix (75) are

$$\lambda_1 = \frac{a_1}{1 + b_2(x^* + y^*)} - k_1 \tag{76}$$

$$\lambda_{2,3} = \frac{-\Phi \pm \sqrt{\Phi^2 - \tau}}{2\theta_x^2\eta_y^2\rho_y^2\xi_x^2}. \tag{77}$$

where Φ and τ are defined by (38) and (39) in the proof of **Theorem 3**, as follows:

$$\Phi = \eta_y\xi_x(\rho_y^2\xi_x\alpha_1\beta_1 x + \theta_x^2\eta_y\gamma_1\sigma_1 y), \tag{78}$$

$$\tau = 4\theta_x^2\eta_y^2\rho_y^2\xi_x^2\alpha_1\gamma_1 xy(\beta_1\sigma_1\eta_y\xi_x - \beta_2\sigma_2\theta_x\rho_y). \tag{79}$$

Since (x^*, y^*) is a stable state of the X-Y system, based on **Theorem 3**, it is clear that τ is positive. Moreover, Φ is always positive. The two eigenvalues λ_2 and λ_3 are negative or have negative real part. Thus, the equilibrium state $(x^*, y^*, 0)$ is a stable state of the X-Y-Z system if

$$\frac{a_1}{1 + b_2(x^* + y^*)} < k_1. \tag{80}$$

□
